# Dosimetric feasibility of moderately hypofractionated/dose escalated radiation therapy for localised prostate cancer with intensity-modulated proton beam therapy using simultaneous integrated boost (SIB-IMPT) and impact of hydrogel prostate-rectum spacer

**DOI:** 10.1186/s13014-022-02025-2

**Published:** 2022-04-01

**Authors:** Dalia Ahmad Khalil, Danny Jazmati, Dirk Geismar, Jörg Wulff, Christian Bäumer, Paul Heinz Kramer, Theresa Steinmeier, Stefanie Schulze Schleitthoff, Sandija Plaude, Martin Bischoff, Stephan Tschirdewahn, Boris Hadaschik, Beate Timmermann

**Affiliations:** 1 Department of Particle Therapy, University Hospital Essen, West German Proton Therapy Centre Essen (WPE), West German Cancer Center (WTZ), German Cancer Consortium (DKTK), Hufelandstraße 55, 45147 Essen, Germany; 2 Department of Urology, University Hospital Essen, University of Duisburg-Essen, Essen, Germany; 3 German Cancer Consortium (DKTK), Essen, Germany

**Keywords:** Proton therapy, Intensity-modulated therapy, Simultaneous integrated boost, Prostate cancer, Hydrogel prostate-rectum spacers

## Abstract

**Purpose:**

To examine the dosimetric feasibility of hypofractionated/dose escalated radiation therapy in patients with localized prostate carcinoma using simultaneous integrated boost intensity-modulated proton beam therapy (SIB-IMPT) in absence or presence of prostate-rectum spacer.

**Methods:**

IMPT technique was implemented in 23 patients with intermediate- and high-risk prostate cancer treated at West German Proton Therapy Centre from March 2016 till June 2018, using SIB technique prescribing 60 GyRBE and 72 GyRBE in 30 fractions to PTV1 (prostate and seminal vesicle) and PTV2 boost (prostate and proximal seminal vesicle), respectively. In 15 patients, a transperineal injection of hydrogel was applied prior to radiotherapy to increase the distance between prostate and rectum. Planning and all treatments were performed with a 120 ml fluid-filled endorectal balloon customised daily for each patient. For each patient, 2 lateral IMPT beams were implemented taking a field-specific range uncertainty (RU) into account. Dose volume histograms (DVH) were analyzed for PTV2, PTV2 with range uncertainty margin (PTV2RU), rectum, bladder, right/left femoral heads, and penile bulb. For late rectal toxicities, the normal tissue complication probabilities (NTCP) were calculated using different biological models. A DVH- and NTCP-based dosimetric comparison was carried out between non-spacer and spacer groups.

**Results:**

For the 23 patients, high-quality plans could be achieved for target volume and for other organs at risk (OARs). For PTV2, the *V*_107%_ was 0% and the D_max_ did not exceed 106.2% of the prescribed dose. The volume PTV2RU covered by 95% of the dose ranged from 96.16 to 99.95%. The conformality index for PTV2RU was 1.12 ± 0.057 and the homogeneity index (HI) was 1.04 ± 0.014. Rectum D_max_ and rectal volume receiving 73–50 Gy could be further reduced for the spacer-group. Significant reductions in mean and median rectal NTCPs (stenosis/necrosis, late rectal bleeding ≥ 2, and late rectal toxicities ≥ 3) were predicted for the spacer group in comparison to the non-spacer group.

**Conclusion:**

Hypofractionated/dose escalated radiotherapy with SIB-IMPT is dosimetrically feasible. Further reduction of the rectal volumes receiving high and medium dose levels (73–50 Gy) and rectal NTCP could be achieved through injection of spacers between rectum and prostate.

## Introduction

Dose-escalated external beam radiotherapy for localised prostate cancer (> 75 Gy) has in multiple trials been proven to lower the risk of biochemical relapse and increase the distant metastasis-free survival with acceptable acute and long-term toxicities [1–3]. Furthermore, hypofractionated radiation therapy (> 2 Gy dose/fx) has been supported in clinical studies for patients with localised prostate cancer due to the assumed low alpha/beta ratio of prostate cancer cells. Several randomised phase II/III trials revealed that hypofractionation yields similar or non-inferior cancer control outcomes, rates of late toxicity, and quality of life results as conventional fractionation [4–12]. According to the most recently published ASTRO, ASCO, and AUA Evidence-Based Guideline, a moderate hypofractionation (2.4–3.4 Gy per fraction, daily, over 4–6 weeks) should be strongly recommended to prostate cancer patients choosing external beam radiation, across all risk groups [13]. A major consideration, or rather limitation, of hypofractionated therapy is the potential increase in the probability of normal tissue complications (NTCPs), and particularly, rectal toxicities. In this context, major attempts are being pursued to increase the therapeutic index; one of them is to offer a highly conformal radiation technique, another to increase the distance between prostate and rectal wall (anterior, lateral, or posterior) either by application of an endorectal balloon, injection of a spacer, or both.

Proton therapy as definitive therapy has been routinely used for localised prostate carcinoma in many centres [14, 15]. With pencil beam scanning (PBS), a proton beam can be magnetically scanned across the target volume, achieving distal and proximal dose conformality. The great potential of PBS is the application of a complex intensity-modulated technique (IMPT), by which the dose can be modulated along the beam axis as well as at the lateral direction of the beam, in an attempt to achieve a maximal sparing of normal tissue while delivering the prescribed dose to target volume in order to maximise the therapeutic ratio [16]. More challenging, however, is the simultaneous integrated boost technique (SIB), by which the overall treatment time can be shortened and a biologically effective dose to the smaller high-risk boost volume can be escalated by hypofractionation. At the same time, the larger low-risk target volume can receive a conventional dose of 2 Gy/fx or even lower. In case of photon therapy using intensity modulated radiation therapy (IMRT), volumetric arc therapy (VMAT), and tomotherapy, some treatment planning studies [17–20] and phase I/II clinical trials [21–23] have been conducted to determine the safety and potential benefit of dose delivery using SIB fractionation. The hypofractionated/dose escalated radiation therapy with SIB technique has not yet been evaluated using proton beam therapy. The aim of this study is to demonstrate the dosimetric feasibility of SIB-IMPT technique and to analyse the dosimetric/NTCP advantages of prostate-rectum spacers in patients with prostate cancer treated with this technique.

## Study design/patients and methods

Since August 2015 a prospective single-centre register study (ProRegPros) evaluating proton therapy for patients with localised prostate cancer has been carried out at the West German Proton Therapy Centreer and since March 2016 we have begun to offer hypofractionated/dose escalated SIB-IMPT as an option to patients with intermediate- and high-risk prostate cancer. Pretreatment staging included prostate-specific antigen (PSA), histologic diagnosis, magnetic resonance imaging (MRI), CT, bone scan, surgical lymphadenectomy for lymph node assessment, or radiologic assessment with MRI, and if applicable PSMA-PET/CT. SIB-IMPT with or without androgen deprivation therapy (ADT) was implemented in 23 patients with cT2b-4 histologically proven prostate adenocarcinoma treated consecutively between March 2016 and June 2018. In 15 patients, a transperineal injection of hydrogel spacer was carried out successfully one week before acquisition of the planning CT to increase distance between prostate and rectum (spacer group). Seven patients refused spacer injection, and one patient had improper implantation of the spacer (non-spacer group). Characteristics are listed in Table 1.

**Table 1:** Patients’ characteristics

Variable	23 Patients No (%)
*Age at diagnosis (years)*	
Range (Mean ± SD)	52–79 (66.65 ± 7.36)
*Prostate volume (cc)*	25.73–109.35 (60.67 ± 21.18)
*PSA at diagnosis (ng/ml)*	
Range (Mean ± SD)	5.03–34.78 (13.1 ± 8.5)
< 10	11 (47.8%)
10–20	7 (30.4%)
> 20	5 (21.7%)
*Gleason score*	
3 + 4	10 (43.5%)
4 + 3	5 (21.7%)
≥ 4 + 4	8 (34.8%)
*T stage*	
cT2b	4 (17.4%)
cT2c	14 (60.9%)
cT3a	2 (8.7%)
cT3b	1 (4.3%)
cT4	2 (8.7%)
*N Stage*	
N0	23 (100%)
N+	0
*ADT*	
Yes	10 (43.5%)
No	13 (56.5%)
*Hydrogel spacer*	
Yes	15 (65.2%)
No	8 (34.8%)

*PSA* prostate-specific antigen, *ADT* androgen deprivation therapy

All patients underwent non-contrasted planning computed tomography (CT) scans with 1 mm axial slice thickness in the supine position and were immobilised with an individualised vacuum cushion and an individualised body thermoplastic cast fixed to the couch. Because of the steep drop in dose beyond the spread-out Bragg peak (SOBP) with subsequent high sensitivity of the dose distribution to the intra-fraction motion of the prostate, we aimed at minimising the prostate motion. A fixed bladder filling protocol (to drink 350 ml on empty bladder 30 min prior to treatment) applied to all patients. For further fixation of the prostate, to increase the distance between prostate and the dorsal rectal wall, and in order to gain a fixed reproducible rectal volume, the planning and treatment were implemented with a 120 ml fluid-filled endorectal balloon customised daily for each patient. For daily pretreatment prostate localisation, three fiducial markers (Visicoil™ 0.5 m × 0.5 cm) were implanted for each patient at the same setting of hydrogel spacer injection using a transperineal approach with transrectal ultrasound guidance. For better target volumes/OARs delineation, each patient underwent a planning-MRI, T1-weighted/T2-weighted images, with and without contrast media. Target volumes were defined on co-registered CT and MRI scans as follow; the gross tumor volume (GTV) was the prostate, and the clinical target volume for low risk volume (CTV1) was defined as the GTV + 5 mm peri-prostatic tissue + 2 cm of the seminal vesicles. In case of extracapsular extension or a cT4 situation, the CTV1 was laterally extended to the pelvic sidewall. The CTV for high risk volume (CTV2) was defined as the GTV + 1 cm of the seminal vesicles. Two planning volumes, PTV1 and PTV2 were generated by adding 5-mm margins in all directions (except for 7 mm expansions at the seminal vesicle region) around CTV1 and CTV2, respectively. PTV1 and PTV2 were treated simultaneously in 30 fractions with 2 dose levels; a dose of 60 Gy (2 Gy/fraction) and 72 Gy (2.4 Gy/fraction) was delivered to the PTV1, and PTV2, respectively. The dose to PTV2 is biologically equivalent to 80.2 Gy in 2 Gy/fx, assuming an α/β ratio of 1.5 for prostate cancer. The rectum was contoured as a solid organ extending from just above the anal verge up to the sigmoid flexure.

IMPT planning and optimisation were performed using the RaySearch’s treatment planning system version 5 (RaySearch Laboratories, Stockholm, Sweden) with pencil beam algorithm [24]. Two lateral-opposed IMPT beams were implemented taking into consideration the range uncertainty, by applying additional distal margin of 3.5% in the proton beam range + 2-mm to the PTV, with subsequent generation of corresponding PTV1RU and PTV2RU. Given the opposing beam arrangement, a non-robust optimization strategy was implemented based on a beam-specific PTV, considering the setup and range uncertainties. For each patient, the calculation of perturbed scenarios for the final plan showed sufficient coverage for setup- and range-uncertainty combinations.

Optimization for each plan was done until fulfilment of the dose distribution requirements and OARs constraints; D_pres(GTV,CTV)_ = 100%, D_95%,(PTV)_ ≥ 95%, D_2%,(PTV2)_ ≤ 107%; RV_73Gy_ < 2%, RV_68.4 Gy_ < 12%, RV_66Gy_ < 20%, RV_62Gy_ < 25%, RV_60Gy_ < 35%, RV_50Gy_ < 50%, RV_40Gy_ < 70%, BV_73Gy_ < 12%, BV_68Gy_ < 20%, BV_66Gy_ < 30%, BV_64Gy_ < 45%, BV_50Gy_ < 60%; femoral head D_max_ < 40 Gy, penile bulb D_mean_ < 50 Gy.

The automatic setting of RayStation was applied for planning in this study, by which the layer separation between two adjacent energy layers equals the energy loss over the 80% level and spot spacing is 1.06 times the average projected, energy dependent sigma.

The following dosimetric parameters were analysed for PTV2 and PTV2RU; D_max_, D_2%,_ D_mean_, D_median_, D_98%_, D_5%,_ D_95%,_ and PTV_95%IDL_ (volume of PTV covered by 95% of the prescribed dose = 68.4 Gy).

For further plan evaluation, the conformality index (CI) and the dose homogeneity index (HI) were collected for PTV2 and PTV2RU;

- The conformality index (CI) was defined as a ratio between reference isodose volume (V_RI_ = volume received 95% of the prescribed dose = 68.4 Gy) and target volume = V_RI_/Volume of PTV
- The HI was defined as a ratio between the dose reached in 5% of the PTV volume and the dose reached in 95% of the PTV volume = D_5%_/D_95%_

For OARs the following parameters were analysed; for rectum; D_max_, D_mean_, D_median_, RV_73Gy_ *(percent of rectal volume received 73 Gy),* RV_72Gy_,RV_70Gy_, RV_68.2Gy_ *(percent of rectal volume received 95% of the prescribed dose)*, RV_766Gy_, RV_65Gy_, RV_62G_, RV_60Gy_, RV_55Gy_, RV_50Gy,_ RV_40Gy_, RV_30Gy_, RV_20Gy,_ and RV_10Gy_; For bladder; D_max_, D_mean_, D_median_, BV_73Gy_ *(percent of bladder volume received 73 Gy)*, BV_72Gy_, BV_70Gy_, BV_68.2Gy_ *(percent of bladder volume received 95% of the prescribed dose)*, BV_65Gy_, BV_60Gy_, BV_55Gy_, BV_50Gy,_ BV_40Gy_, BV_30Gy_, BV_20Gy_ and BV_10Gy_*;* for right and left femoral heads; D_max_, and D_mean_; and for penile bulb; *D*_*mean*_*.*

Further analysis was performed for the rectum as a solid organ, distinguishing between the spacer group (15 patients) and the non-spacer group (8 patients).

For the NTCP calculation, different biological models were used for the rectum; the Poisson-LQ model for necrosis/stenosis with *D*_50_ = 80 Gy, *γ* = 2.2, S = 1, and *α*/*β* = 3 [25]; the Layman Kutcher Burman (LKB) model for late rectal bleeding ≥ 2 with *D*50 = 81.8 Gy, *γ* = 3, m = 0.22, n = 0.29, and *α*/*β* = 3 [26]; and LKB model for late effects grade ≥ 3 with *D*_50_ = 80 Gy, m = 0.15, n = 0.06, and *α*/*β* = 3.9 [27].

The separation distance between posterior surface of the prostate and anterior rectal wall (D_P-R_) was measured for all patients at midline in T2-weighted planning MRI sequence at apex, mid-zone, and base and then correlated with rectal dose distribution and NTCP values.

All results were described as range and mean ± standard deviation (± SD). The Wilcoxon-Mann–Whitney-Test was used to compare continuous data between the spacer group and the non-spacer group non-parametrically. The associations between two continuous variables were quantified using Pearson correlation. Statistical analysis was done using the IBM SPSS Statistics programme V22.

## Results

### Plan quality

For all patients, planning with the SIB-IMPT technique resulted in good dose distribution quality (Table 1). All plans fulfilled the prescribed doses to the targets; D_95%,(PTV)_ ≥ 95%, and D_2%,(PTV)_ ≤ 107%). For PTV2, the *V*_107%_ = 0%. The D_max_ did not exceed 76.5 Gy (106.2% of the prescribed dose). The target dose coverages were generally good for all plans. The mean PTV_95%IDL_ was 99.62% for PTV2 and 98.84% for PTV2RU. The CI for PTV2 showed a mean value of 1.27 (ranging from 1.27 to 1.63). For PTV2RU we recorded lower CI with a mean of 1.12 (range 1.02–1.21). Regarding dose homogeneity, SIB-IMPT generated homogenous dose distribution with HI mean of 1.02 for PTV2 and 1 for PTV2RU (Table2).

**Table 2:** Dose–volume histogram results for PTV2RU

	Range	Mean ± SD
Volume (cc)	113.2–329.25	194.58 ± 63.04
D_max_ (Gy)	73.2–76.5	74.39 ± 0.86
D_2%_ (Gy)	72.7–74.0	73.22–0.31
D_mean_ (Gy)	71.54–72.06	71.78 ± 0.11
D_median_ (Gy)	71.82–72.14	71.94 ± 0.1
D_95%_ (Gy)	68.85–71.03	69.92 ± 0.59
D_98%_ (Gy)	66.68–70.16	69.0 ± 0.92
PTV_95%IDL_ (%)	96.16–99.95	98.84 ± 1.21
CI	1.02–1.21	1.12 ± 0.06
HI	1.00–1.06	1.04 ± 0.01

RU = range uncertainty*,* CI = conformity index = V_RI_/Volume of PTV, HI = dose homogeneity index = D_5%_/D_95%_

**Fig. 1:**
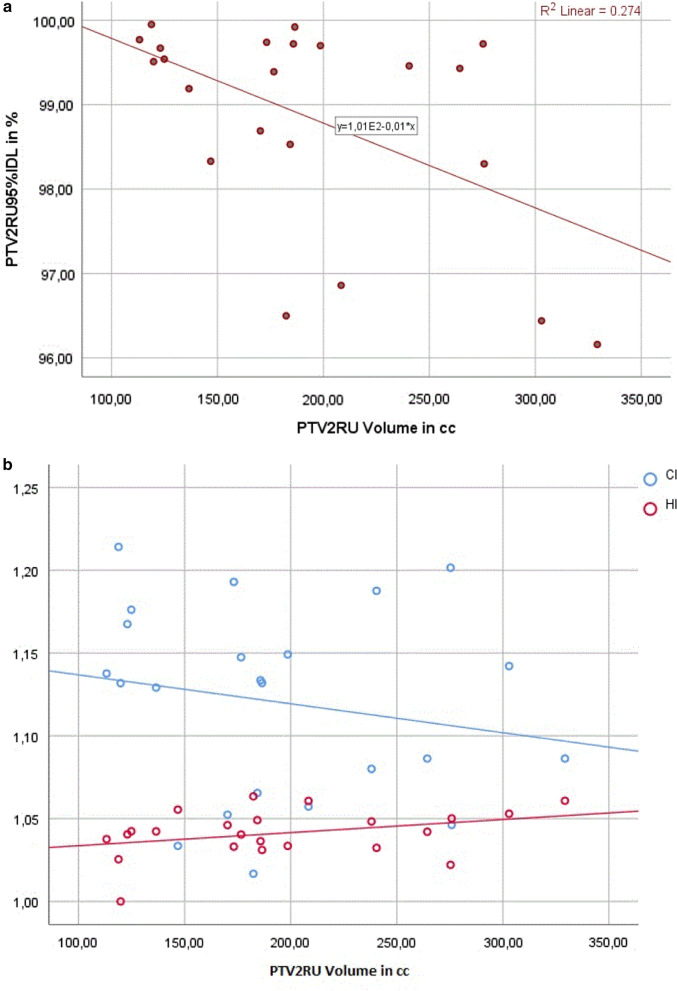
**a** Coverage of PTV2RU to volume of PTV2RU; significant negative correlation (*P* = 0.027). **b** CI to volume of PTV2RU, and HI to volume of PTV2RU; no correlation

Regarding PTV2RU coverage, a statistically significant negative correlation was found between the volume of PTV in cc and the percentage of PTV_95%IDL_ (R =  − 0.461, R^2^ = 0.21 *P* = 0.027), which means that for smaller PTV volumes higher coverage of PTV2 could be reached. No significant correlation between the CI and corresponding volume of PTV could be observed (R =  − 0.169, *P* = 0.441). Similarly, no significant correlation between the HI and corresponding volume of PTV could be reported (R = 0.298, *P* = 0.167). The linear correlation between Volume of PTV2RU in cc and PTV2RU coverage, CI, and HI are shown in Fig. 1a, b.

### Organs at risk

Table 3 summarizes the DVH results for all OARs. The dose distribution satisfied all physical constraints. The mean of maximal dose delivered to the bladder was 73.5 Gy, and the BV72Gy ranged from 0.03 to 11.37%. For all patients, rectum D_max_ ranged between 71.02 and 76 Gy. The RV_72Gy_ ranged between 0 and 6.66%, the mean rectal volumes received 70 Gy, 68.4 Gy, 65 Gy, 60 Gy, 50 Gy, and 40 Gy were 4.6%, 6.02%, 8.56%, 12.14%, 19.16%, and 24.86%, respectively.

**Table 3:** Dose–volume histogram results for OARs

OARs	Range	Mean ± SD
*Bladder*		
Volume	81.2–477.8	231.8 ± 131.8
D_max_	72.20–75.00	73.51 ± 0.70
D_mean_	6.61–38.86	20.81 ± 9.57
D_median_	0.16–39.69	10.85 ± 11.91
BV_73Gy_	0–4.0	0.74 ± 1.12
BV_72Gy_	0.03–11.37	4.14 ± 3.37
BV_70Gy_	0.61–17.78	7.85 ± 5.51
BV_68.4 Gy_	0.89–20.69	9.34 ± 6.36
BV_65Gy_	1.53–24.83	11.87 ± 7.38
BV_60Gy_	2.50–29.69	15.07 ± 8.78
BV_55Gy_	3.48–36.65	18.05 ± 10.13
BV_50Gy_	4.67–40.65	20.62 ± 11.03
BV_40Gy_	6.73–49.67	25.45 ± 12.83
BV_30Gy_	9.15–58.30	30.24 ± 14.43
BV_20Gy_	11.95–67.98	35.79 ± 16.31
BV_10Gy_	16.12–80.30	43.49 ± 18.61
*Rectum*		
Volume	135.2–231.1	179.3 ± 27.0
D_max_	71.02–76.00	73.49 ± .99
D_mean_	9.73–36.39	20.84 ± 6.14
D_median_	1.60–38.41	9.50 ± 8.34
RV_73Gy_	0.00–1.53	0.31 ± 0.43
RV_72Gy_	0.00–6.66	1.94 ± 1.69
RV_70Gy_	0.05–10.72	4.64 ± 3.28
RV_68.4 Gy_	0.15–12.64	6.03 ± 3.89
RV_66Gy_	0.37–15.83	7.85 ± 4.70
RV_65Gy_	0.51–17.69	8.56 ± 5.0
RV_62Gy_	1.05—23.25	10.68 ± 5.92
RV_60Gy_	1.82–26.75	12.14 ± 6.29
RV_55Gy_	3.12–33.46	15.90 ± 7.03
RV_50Gy_	4.57–38.92	19.16 ± 7.71
RV_40Gy_	8.26–48.65	24.87 ± 8.97
RV_30Gy_	12.61–56.85	30.54 ± 9.96
RV_20Gy_	18.25–64.97	37.01 ± 10.83
RV_10Gy_	26.67–74.97	46.13 ± 11.63
*Femoral head*		
Right D_max_	30.60–47.60	34.3 ± 4.28
Right D_mean_	17.26–31.26	26.73 ± 3.5
Left D_max_	30.60–50.00	34.38 ± 4.16
Left D_mean_	18.14–31.35	27.02 ± 3.59
*Penile bulb*		
D_mean_	2.96–61.43	23.18 ± 19.32

*Dose in Gy; BV_XGy_ = Percentage of bladder volume received X dose; RV_XGy_ = Percentage of rectal volume received X dose

### Dosimetric comparison between non-spacer group and spacer group

**Fig. 2:**
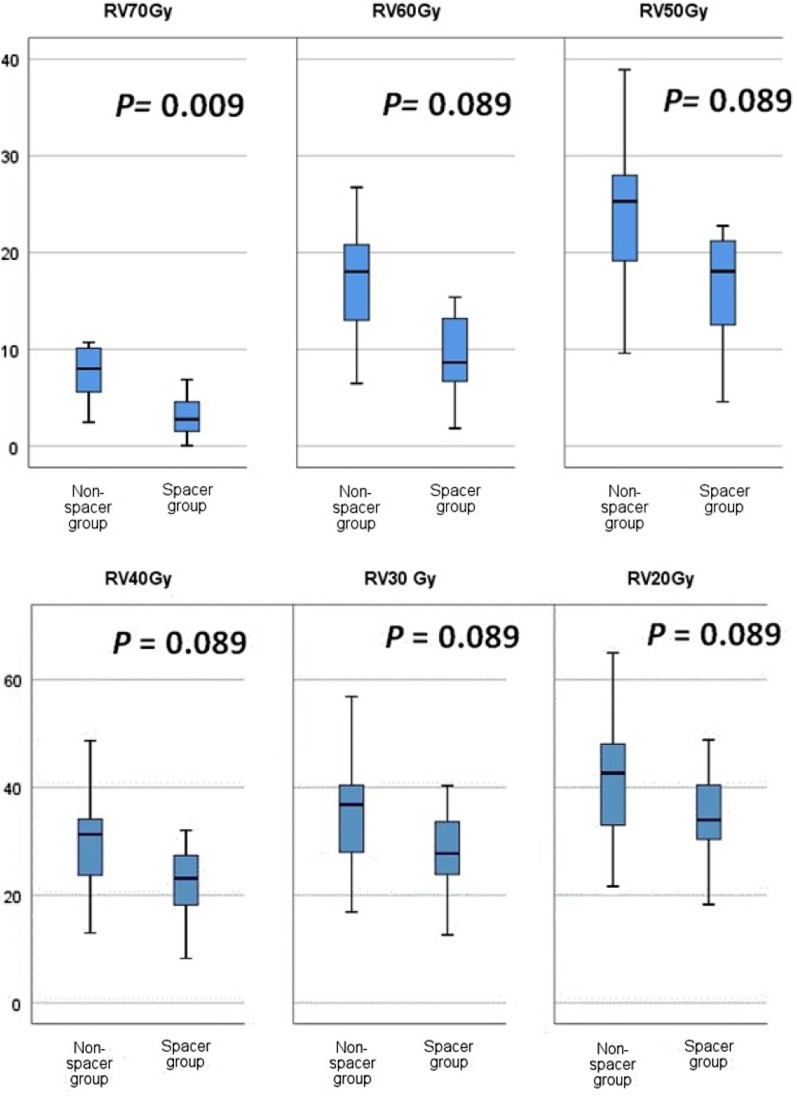
Box plot comparing the median (range) of RV_70Gy_, RV_60Gy_, RV_50 Gy_, RV_40Gy_, RV_30Gy_, and RV_20Gy_ for non-spacer group vs. spacer group

There was no statistically significant difference between the spacer group and the non-spacer group regarding the volume of the prostate (*P* = 0.333). The average of the maximum doses received by rectum was lower for spacer group in comparison to non-spacer group. Also the mean values of RV_73Gy_ RV_72Gy_*,* RV_70Gy_, RV_68.2Gy_, RV_66Gy_, RV_65Gy_, RV_62G_, RV_60Gy_, RV_55Gy_, and RV_50Gy_ were significantly reduced in spacer group compared to non-spacer group. By analysis of mean values of rectal volumes which received 40 Gy, 30 Gy, 20 Gy, and 10 Gy, we found that spacer group had lower values than non-spacer group, but these differences did not reach statistical significance (Table 4). By median comparison, the Boxplot analysis visualizes a statistical significant reduction in median of RV_70Gy_ for spacer group vs. non-spacer group, *P* = 0.039. By further analysis of median values of RV_60Gy_, RV_50G_, RV_40Gy_, RV_30Gy_, and RV_20Gy,_ we did not find any statistical significant differences between the 2 groups (Fig. 2).

**Table 4:** Dosimetric evaluation for rectum according to spacer

*	Non-spacer group (8 patients)	Spacer group (15 patients)	*P value*
Prostate volume (cc)	66.48 ± 22.07 (41.30–109.35)	57.57 ± 20.78 (25.73–95.96)	0.333
Rectal volume (cc)	191.7 ± 30.9 (154.9–231.1)	172.8 ± 23.1 (135.2–206.9)	0.131
Rectum D_max_	74.15 ± 0.87 (73.00–76.0)	73.15 ± 0.89 (71.02–74.0)	**0.013**
Rectum D_mean_	24.10 ± 7.56 (12.66–36.39)	19.1 ± 4.6 (9.73–25.75)	0.065
Rectum D_median_	13.44 ± 11.88 (2.13–38.41)	7.4 ± 5.02 (1.6–18.59)	0.238
RV_73Gy_	0.67 ± 0.58 (0.05–1.53)	0.12 ± 0.13 (0–0.42)	**0.007**
RV_72Gy_	3.34 ± 1.89 (1.12–6.66)	1.19 ± 1.0 (0.00–3.04)	**0.005**
RV_70Gy_	7.56 ± 2.97 (2.45–10.72)	3.078 ± 2.23 (0.05–6.87)	**0.001**
RV_68.4 Gy (95% of dose)_	9.52 ± 3.41 (3.32–12.64)	4.17 ± 2.71 (0.15–8.8)	**0.002**
RV_66Gy_	11.95 ± 4.27 (4.27–15.83)	5.67 ± 3.31 (0.37–10.86)	**0.003**
RV_65Gy_	12.90 ± 4.57 (4.93–17.69)	6.25 ± 3.52 (0.51–11.63)	**0.003**
RV_62Gy_	15.51 ± 5.86 (5.76–3.25)	8.1 ± 4.18 (1.05–14.08)	**0.005**
RV_60Gy_	17.11 ± 6.12 (6.46–26.75)	9.49 ± 4.32 (1.82–15.40)	**0.005**
RV_55Gy_	20.95 ± 8.1 (7.95–33.46)	13.22 ± 4.74 (3.12–18.74)	**0.016**
RV_50Gy_	24.18 ± 9.20 (9.6–38.92)	16.49 ± 5.38 (4.57–22.76)	**0.019**
RV_40Gy_	29.96 ± 11.1 (12.97–48.65)	22.15 ± 6.48 (8.26–32.02)	0.056
RV_30Gy_	35.49 ± 12.51 (16.84–56.85)	27.91 ± 7.49 (12.61–40.32)	0.065
RV_20Gy_	41.75 ± 13.72 (21.62–64.97)	34.48 ± 8.39 (18.25–48.8)	0.149
RV_10Gy_	50.42 ± 14.65 (29.74–74.97)	43.85 ± 9.44 (26.67–59.37)	0.231

Significance *P* value < 0.05 (Bold)

*Dose in Gy; RV_X Gy_ = Percentage of rectal volume received X dose; all result are presented in mean ± SD (range)

For all patients, the mean D_P-R_ at apex, mid-zone, and base were 10.67 ± SD 2.91 (range 6.6–16.4 mm), 12.93 ± SD1.97 (range 9.4–15.9 mm), and 12.07 ± SD 3.09 (range 6–16 mm), respectively. The maximal D_P-R_ at all sections was 10.32 ± SD 5.38 (2.5–16.4 mm). The maximal D_P-R_ correlated negatively with Rectum D_max_ (R^2^ = 0.22, *P* = 0.024), Rectum D_mean_ (R^2^ = 0.21, *P* = 0.029), Rectum D_median_ (R^2^ = 0.18, *P* = 0.044), RV_73Gy_ (R^2^ = 0.36, *P* = 0.002), RV_72Gym_ (R^2^ = 0.316, *P* = 0.005), RV_70Gy_ (R^2^ = 0.38, *P* = 0.002), RV_68.4Gy_ (R^2^ = 0.39, *P* = 0.001), RV_66Gy 4Gy_ (R^2^ = 0.38, *P* = 0.002), RV_65Gy 4Gy_ (R^2^ = 0.39, *P* = 0.001), RV_60Gy 4Gy_ (R^2^ = 0.345, *P* = 0.003), RV_55Gy 4Gy_ (R^2^ = 0.31, *P* = 0.006), RV_50Gy 4Gy_ (R^2^ = 0.27, *P* = 0.011), RV_40Gy 4Gy_ (R^2^ = 0.23, *P* = 0.021), RV_30Gy 4Gy_ (R^2^ = 0.192, *P* = 0.037). No correlation was observed between D_P-R and_ RV_20Gy_ or RV_10Gy._

For the non-spacer group, the mean D_P-R_ at apex, mid-zone, and base were 1.45 ± SD 0.27 (range 1.2–2 mm), 2.4 ± 1.3 (range 91.1–4.2 mm), and 2.9 ± SD 0.68 (range 2–4.1 mm), respectively. The maximal D_P-R_ at all sections was 3.4 ± SD 0.69 mm (range 2.5–4.2 mm). For the spacer group, the mean D_P-R_ at apex, mid-zone, and base were 10.67 ± SD 2.91 (range 6.60–16.40 mm), 12.93 ± 1.97 (range 9.40–15.90 mm), and 12.07 ± 3.09 (range 6.00–16.00 mm), respectively. The maximal D_P-R_ at all sections was 14.01 ± SD 1.76 (10.1–16.4 mm). By analysis of each group separately, we could not observe any correlation between the maximal D_P-R_ achieved and rectal doses except for spacer group a negative correlation between the Rectum D_median_ and the maximal D_P-R_ (R^2^ = 0.28, *P* = 0.044) could be reported.

### NTCP calculation

**Fig. 3:**
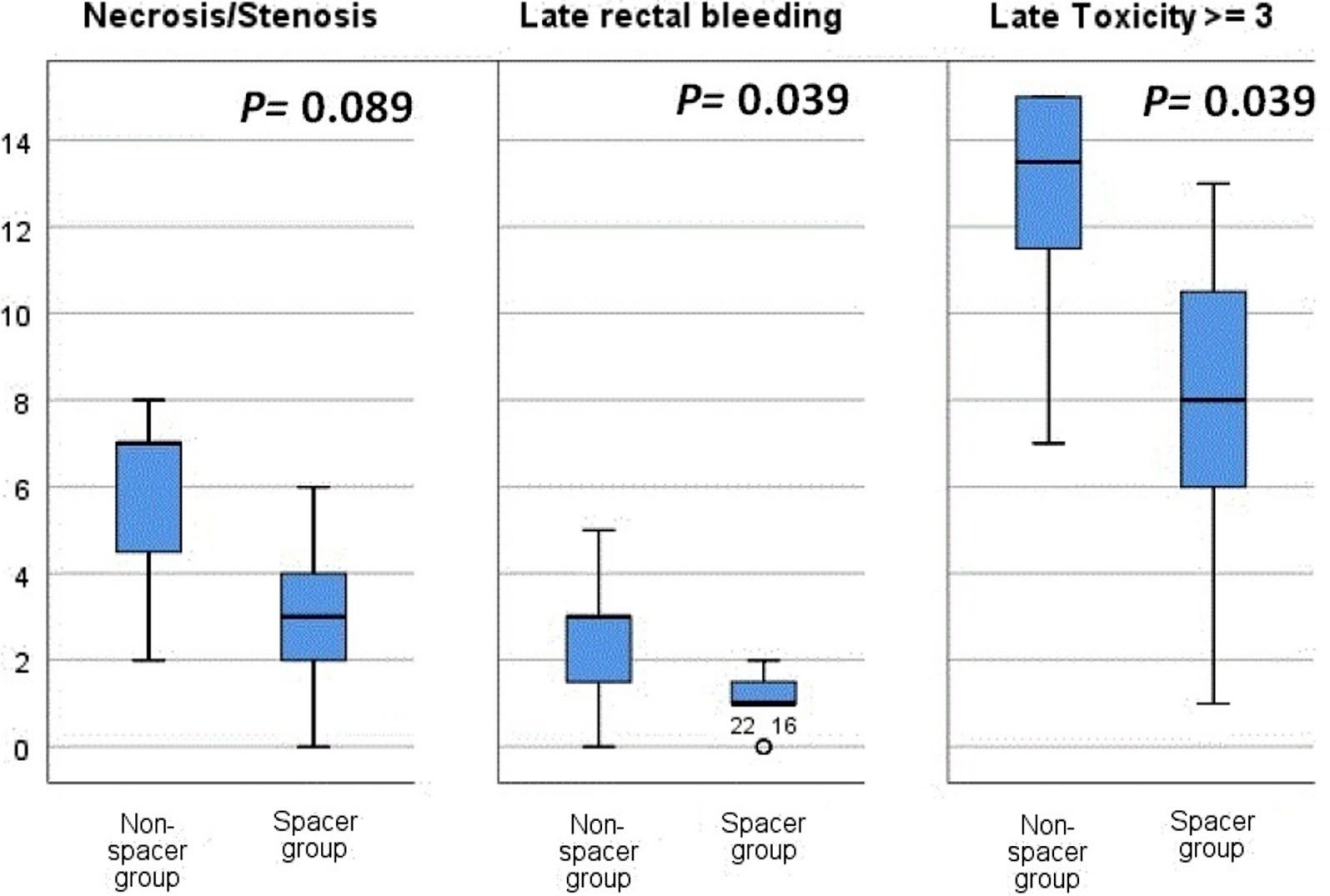
Box plot comparing median (range) of the predicted rectal NTCP rates for necrosis/stenosis, late bleeding, and late toxicity ≥ 3 for non-spacer group vs. spacer group

For all patients the SIB-IMPT plan resulted in acceptable NTCP rates of late rectal toxicities. The application of spacer provided significantly lower NTCPs. Significant decreases in mean (*P* = 0.005) of late rectal necrosis/Stenosis, in mean (*P* = 0.016) and median (*P* = 0.039) of late rectal bleeding, and in mean (*P* = 0.002) and median (*P* = 0.039) of late rectal toxicities ≥ 3 were predicted for spacer group vs. non-spacer group (Table 5, Fig. 3). The maximal D_P-R_ strongly negatively correlated with NTCP values of late rectal necrosis/Stenosis (R^2^ = 0.347, *P* = 0.003), of late rectal bleeding (R^2^ = 0.3, *P* = 0.007), and of late rectal toxicities ≥ 3 (R^2^ = 0.32, *P* = 0.005).

**Table 5:** Predicted late rectal toxicity rates using spatial NTCP models

End point *	Whole group (23 patients)	Non-spacer group (8 patients)	Spacer group (15 patients)	*P value*
*Necrosis/stenosis*				
Mean ± SD	3.9 ± 2.37%	5.87 ± 2.17%	2.87 ± 1.77%	0.005
Range	(0–8%)	(2–8%)	(0–6%)	
*Late rectal bleeding* ≥ *3*				
Mean ± SD	1.56 ± 1.24%	2.5 ± 1.51%	1.067 ± 0.7%	0.016
Range	(0–5%)	(0–5%)	(0–2%)	
*Late toxicity* ≥ *3*				
Mean ± SD	9.43 ± 4.15%	12.75 ± 2.87%	7.67 ± 3.66%	0.002
Range	(1–15%)	(7–15%)	(1–13%)	

*Poisson-LQ model for Necrosis/stenosis [25], LKB model for late rectal bleeding ≥ 3 [26], and LKB model for late effects ≥ 3[27]

## Discussion

Moderate hypofractionation as a tool for dose escalation has gained widespread acceptance for patients with localised prostate cancer. Therefore it has become important in the West German Proton Therapy Centre to prospectively evaluate proton therapy in this context. In our feasibility study we analysed the dosimetric parameters of an optimised SIB-IMPT plan for dose escalation to 72 Gy in a hypofractionated pattern with 2.4 Gy dose/fx to the boost volume. The demonstrated results show that treatment planning with SIB-IMPT is possible and could reach the preset dose prescriptions for the PTV with good CI for all plans, while sparing the OARs. One of the drawbacks during the planning of proton therapy is the addition of RU margins to the PTV, which means that a higher volume of healthy tissue is encompassed in the radiation field. For all plans, we applied a simple beam configuration with 2 lateral opposed beams and applied the RU margins at the distal end of the beams (lateral to the prostate, and not ventral or dorsal) with avoidance of any beam direction which can stop at the rectum or at the bladder. By analysis of the PTV2RU, we reported excellent CI results with a mean of 1.12 ± 0.057 (range 1.02–1.21). Also, we could report good PTV coverage and homogeneity results.

For all patients SIB-IMPT planning could reduce the bladder and rectal volume exposed to high radiation doses. Regarding bladder dosimetry, it has been proven in multiple clinical trials that the maximum dose to the bladder and the bladder volume receiving 70 Gy are predictors for grade 2 genitourinary toxicities [28, 29]. More recently it has been proven that not only high-dose volumes can contribute to late toxicities but also mid-dose volumes (60–40 Gy) [30]. Macias et al. [31] in their study tested a 2.6 Gy/fraction to a total dose of 67.6 for low-risk (biologically equivalent to 79 Gy in 2 Gy/fx) and 70.2 Gy for intermediate–high-risk (biologically equivalent to 82 Gy in 2 Gy/fx) over 5.2–5.4 weeks, and found that BV_65Gy_ is associated with an increased risk of genitourinary complications (*P* = 0.017). Beckendorf et al. published the late toxicity results of the French GETUG 06 randomised trial comparing conventional fractionation to 70 Gy and 80 Gy for localised prostate cancer. The authors found that bladder D_max_ > 75 Gy (*P* = 0.0064) and 50% bladder volume receiving more than 44.7 Gy (*P* = 0.04) are associated with grade ≥ 2 late urinary toxicity [29]. In our study, the average of the bladder D_max_ was 73.51 ± SD 0.70 Gy and assuming that the bladder has an α/β-level of 5 Gy, the 73.51 Gy D_max_ mean in 30 fractions would be biologically equivalent to 78.2 Gy in 2 Gy/fx. Furthermore, the mean BV_70Gy_ in our study was 7.85 ± SD 5.51% and the mean BV_65Gy_ was 11.87 ± SD 7.38%.

The rectal dose-volume relationship with late rectal toxicity has been the scope of numerous trials. Ballare et al. [32] applied conventional fractionation with 74 Gy in 2 Gy/fx and reported that RV_70Gy_ influenced the occurrence of late rectal grade 2 toxicity. Storey et al. [33] also tested conventional fractionation to 70 Gy or 78 Gy in 2 Gy/fx. For the 78 Gy arm, the authors found that patients with RV_70Gy_ more than 25% had a 5-year risk of grade 2 or higher complications of 37% compared to 13% for patients with 25% or less (*P* = 0.05). Someya et al. [34] in their study applied 70 Gy in 35 fractions using 3-D conformal radiation therapy, or 76 Gy in 38 fractions using IMRT, and demonstrated in the multivariate analysis that patients with RV_65Gy_ ≥ 17% had a significantly increased risk of grade 2/3 rectal bleeding (*P* = 0.032). Moving to the hypofractionation era, Pervez et al. used a hypofractionated dose prescription of 68 Gy in 25 fractions (2.72 Gy/fx) to the prostate and to the proximal seminal vesicle and found that RV_60Gy_ correlated with the rectal toxicity [35]. At our centre, an endorectal balloon is regularly used for each prostate patient to daily obtain a reproducible and fixed volume of rectum and in an attempt to increase the distance between the dorsal rectal wall and the prostate. For all patients, the rectum D_max_ was 73.49 ± 0.99 Gy and assuming that the rectum has an α/β-level of 5 Gy, the 73.49 Gy D_max_ mean in 30 fractions would be biologically equivalent to 77.8 Gy in 2 Gy/fx. We could keep the range of RV_70Gy_ between 0.05 and 10.72% (mean 4.64 ± SD 3.28%), and the RV_65Gy_ ranged between 0.51 and 17.69% (8.56 ± 5.0%). In this study, we reported acceptable NTCP results with mean of 3.9% for necrosis/stenosis, 1.56% for late rectal bleeding, and 9.43% for late toxicity ≥ 3.

Strom et al. proved that hydrogel rectum spacers could move the rectum away from the prostate by an average of 12 mm, leading to a significant reduction in rectal radiation doses for patients treated with high dose rate Brachytherapy and IMRT [36]. Mariados et al. conducted a multicenter randomized controlled trial on 222 prostate cancer patients treated with IMRT to 79.2 Gy in 1.8-Gy/fx to test the dosimetric and clinical effects of spacer application. The authors found the postspacer plans had a significant reduction in mean RV_70Gy_ compared to prespacer plans (12.4–3.3%, *P* < 0.0001) [37]. In the German IPI trial, 92 patients with localised prostate cancer were randomised to receive either proton therapy or carbon ion therapy to a total dose of 66 Gy in 20 fractions. Preliminary acute toxicity and quality of life results with median follow-up of 22.3 months showed that hypofractionation is feasible with 2 patients treated with protons developed grade 3 rectal fistulas. The authors reported grade 1 radiation proctitis in 12.1% and grade 2 in 5.5% of patients. In this trial, it was planned to inject hydrogel spacers for all patients, but because of rectal wall hydrogel spacer infiltration with consecutive occurrence of 2 rectal fistulas, the investigators stopped insertion of spacer gel [38]. In this study, in 15 patients we attempted to further distance the anterior rectal wall from the prostate by implantation of absorbable hydrogel rectum spacers. In concurrence to the IPI study, we did not observe any leakage of hydrogel into the rectal wall or into the rectal lumen.

The comparative analysis has proven that the hydrogel spacers could successfully reduce the rectal volumes exposed to high/medium radiation doses. As demonstrated, we found that the implantation of rectum spacers has significantly reduced the rectal D_max_ by 1 Gy (*P* = 0.013), and also reduced the mean RV_72Gy_ from 3.34 to 1.19%, (*P* = 0.005), and mean RV_70Gy_ from 7.56 to 3.1% (*P* = 0.001). Further reduction in rectal volumes received 68.4 Gy, 66 Gy, 65 Gy, 62 Gy, 60 Gy, 55 Gy, and 50 Gy could be also reported. Furthermore, a negative correlation could be observed between the maximal D_P-R_ and Rectum D_max_, Rectum D_mean_, Rectum D_median_, and rectal volumes received doses between 73 and 30 Gy.

Recently, Vanneste et al. [39] reported a statistically significant gain in NTCP, especially grade 2 late rectal bleeding and subjective sphincter control when using hydrogel rectum spacers in prostate cancer patients receiving IMRT. In this study, we reported a significant benefit of space application in reducing the mean and median of rectal NTCPs. Application of spacer reduced the mean NTCP value for late rectal toxicities from 5.87 to 2.87% for necrosis/stenosis, from 2.5 to 1.07% for late rectal bleeding ≥ 3, and from 12.75 to 7.67% for late toxicity ≥ 3. Moreover a strong negative correlation could be observed between the maximal D_P-R_ and NTCP values of late rectal necrosis/Stenosis, late rectal bleeding, and late rectal toxicities ≥ 3.

## Conclusion

In conclusion, the SIB-IMPT technique is dosimetrically feasible and resulted in high-quality proton beam plans. For healthy tissue sparing we applied two lateral opposed beams, but in the context of hypofractionated/dose escalated radiation, we still need a narrower PTV margin to reach stricter dose constraints for the bladder D_max_ and the rectum D_max._ Most dose escalation trials for prostate cancer patients used a PTV margin of 5 mm in all dimensions and only 3 mm posteriorly. Our local guidelines for PTV generation by adding as 5-mm margins in all directions except of 7 mm expansions at the seminal vesicle region has been based on a retrospective study which was carried out in our centre to estimate the effect of intra‐ and interfractional organ motion on the resulting dose distribution by prostate cancer treatment using PBS [40] and based on the fact that the prostate position can be verified and corrected by using the markers, while no markers are available to correct the position of the seminal vesicle. A smaller PTV margin should be tested in the future. Application of prostate-rectum spacer is effective in sparing the rectum and leads to better NTCP results for late rectal toxicities. Currently, a clinical prospective phase II study of moderate hypofractionation (HypoPros; DRKS00011912) is under way at our center. A long follow-up will generate greater clinical evidence for SIB-IMPT technique, also taking into account the late toxicities to be matched with the NTCP models.

## Data Availability

All data and materials can be accessed via DAK, in compliance with data protection guidelines.

## Abbreviations

SIB-IMPT: Intensity-modulated proton beam therapy using simultaneous integrated boost
PTV: Planning target volume
RU: Range uncertainty
DVH: Dose volume histograms
NTCP: Normal tissue complication probabilities
OAR: Organs at risk
CI: Conformality index
PBS: Pencil beam scanning
IMPT: Intensity-modulated technique
SIB: Simultaneous integrated boost technique
IMRT: Intensity modulated radiation therapy
VMAT: Volumetric arc therapy
PSA: Prostate-specific antigen
MRI: Magnetic resonance imaging
ADT: Androgen deprivation therapy
CT: Computed tomography
SOBP: Spread-out Bragg peak
GTV: Gross tumor volume
CTV: Clinical target volume
LKB: Layman Kutcher Burman
